# Supplementary material for: Mercury-methylating bacteria are associated with copepods: A proof-of-principle survey in the Baltic Sea
Source: PLoS One. 2020 Mar 16;15(3):e0230310. doi: 10.1371/journal.pone.0230310 (PMC7075563; doi:10.1371/journal.pone.0230310)
Supplement: S1 Text — (PDF) [file pone.0230310.s001.pdf]

Supporting Information

Mercury-methylating bacteria are associated with copepods: a proof-of-principle survey in the Baltic Sea

Elena Gorokhova*, Anne L. Soerensen and Nisha H. Motwani

Department of Environmental Science and Analytical Chemistry, Stockholm University, SE-10691 Stockholm, Sweden

*Corresponding author:
Elena Gorokhova, elena.gorokhova@aces.su.se

S1 Text 1. Laboratory procedures

DNA extraction. Each sample was transferred into a 1.5-mL centrifuge tube containing 40 μL Tris buffer and approximately 80 μg of acid-washed glass beads (≤ 106 μm; Sigma-Aldrich, MO, USA). The sample was homogenized for 2 × 20 s in a FastPrep®-24 Instrument (MP Biomedicals, CA, USA), and 25 μL of the homogenate were transferred to a new tube. Following addition of 50 μL 10% Instagene Chelex (Bio-Rad), the sample was incubated for 30 min at 105°C [1]. After centrifugation (12 000 *g*, 5 min), the supernatant (20 μL) was transferred to a new tube and stored at 4°C for 2–3 days before the qPCR analysis. This protocol was found to perform well for analysis of prokaryotes in zooplankton guts [2]. The DNA concentration was quantified using a PicoGreen™ double-stranded DNA quantification kit (Life Technologies) and a microplate spectrofluorometer (FLUOstar Optima, BMG LabTechnologies). The DNA quantities recovered from zooplankton guts are presented in S1 Table.

qPCR standards. Following the protocol of Christensen and co-workers [3], three main clades, *Deltaproteobacteria*, *Firmicutes*, and *Archaea*, were considered as potential targets for *hgcA* gene that encodes a putative corrinoid protein [4]. For each clade, a synthetic DNA oligonucleotide comprising the clade-specific target sequence was constructed using a representative strain, *Dv. desulfuricans* for *Deltaproteobacteria*, *Df. metallireducens* for *Firmicutes*, and *Ml. hollandica* for *Archaea* (S1 Table). Each synthetic gene was assembled and cloned into pMA-T plasmid using SfiI and SfiI cloning sites by Invitrogen (Life Technologies). The plasmid DNA was purified from transformed bacteria (*E. coli*, K-12) and concentration

was determined by UV spectroscopy. The final construct was verified by sequencing, and the congruence within the restriction site was 100%. The standards were loaded in duplicates, in five-step tenfold serial dilutions, 1.5×10^6 to 1.5×10^2 gDNA copies per reaction (S2 Table, S2 Fig).

qPCR setup. The qPCR conditions followed existing protocol [3] as summarized in S4 Table. In brief, 6 µL of extracted DNA was loaded in triplicate to 48-well plate plates and dried at 50 °C for 30 min, followed by the addition of a 20 µL of the clade-specific master mix. For all qPCRs, the fluorescent signal was acquired after each extension step and the reaction was limited to 30 cycles to minimize non-specific amplification (Fig S2); this cutoff value was recommended in the original protocol [3]. The melt curve protocol included annealing at the extension temperature and melting at a ramp rate of 0.5 °C/5 s up to 95 °C, with the fluorescent signal acquired continuously during the melt curve. For all amplicons, only one informative peak was detected (S3 Fig), including the spike test samples. No-template controls (NTC) were included in each plate, and in no cases did they produce a positive amplification.

Spike test. When samples were found to produce consistently negative amplification results (cladocerans: all samples; copepods: samples tested for *hgcA* genes of Archaea), we evaluated the ability of the host DNA to inhibit the PCR reaction. A small amount of the *Dv. desulfuricans* standard (1.5×10^4 DNA copy number) was added to a pooled cladoceran sample, whereas *Ml. hollandica* standard (1.5×10^5 DNA copy number) was added to a pooled copepod sample (*E. affinis* and *L. macrurus*; 50:50); these mixtures were used in triplicate as templates in the respective real-time PCR assays. The Ct values of the spiked samples and the corresponding standards run within the dilution series on the same plate were compared using an unpaired t-test. The cladoceran samples spiked with *Dv. desulfuricans* were statistically indistinguishable from the standard (t = 1.225 df = 4, $p > 0.2$), whereas for copepod sample spiked with *Ml. hollandica*, the observed 1% inhibition was marginally significant (t = 2.214, df = 4, $p > 0.09$). Therefore, we concluded that the lack of the *hgcA* amplification in the test samples was not related to the inhibitory properties of the host DNA.

References

1. Giraffa G, Rossetti L, Neviani E. An evaluation of Chelex-based DNA purification protocols for the typing of lactic acid bacteria. J Microbiol Methods 2000;42: 175–184.
2. Motwani NH, Gorokhova E. Metazooplankton grazing on picocyanobacteria as inferred from molecular diet analysis. PLoS ONE 2013;8: e79230

3. Christensen GA, Wymore AM, King AJ, Podar M, Hurt RA, Santillan EU, et al. Development and Validation of Broad-Range Qualitative and Clade-Specific Quantitative Molecular Probes for Assessing Mercury Methylation in the Environment. Appl Environ Microbiol. 2016;82: 6068-6078.
4. Parks JM, Johs A, Podar M, Bridou R, Hurt RA Jr, Smith SD, et al. The genetic basis for bacterial mercury methylation. Science 203;339: 1332-1335.
5. Vermeulen J, Pattyn F, De Preter K, Vercruysse L, Derveaux S, et al. External oligonucleotide standards enable cross laboratory comparison and exchange of real-time quantitative PCR data. Nucl Acids Res. 2009;37: e138.
