# Supplementary material for: Mercury-methylating bacteria are associated with copepods: A proof-of-principle survey in the Baltic Sea
Source: PLoS One. 2020 Mar 16;15(3):e0230310. doi: 10.1371/journal.pone.0230310 (PMC7075563; doi:10.1371/journal.pone.0230310)
Supplement: S1 Table — (PDF) [file pone.0230310.s002.pdf]

S1 Table. DNA yield for zooplankton gut samples.

Data are shown as mean and range (min-max) values for each species and station. In all samples, the mean A260/280 ratio of DNA was 1.78 (range: 1.64-1.85). Observe that this DNA is composed of the DNA from different sources: algal food, zooplankton gut epithelium, and gut microbiota. NS – not sampled.

**Species/stations**	**DNA, ng ind^{-1}**			
	**H4**	**BY31**	**F64**	**US5b**
	Copepoda			
Acartia bifilosa	NS	8.2; 6.4-10.1	11.1; 5.7-12.3	9.3; 4.7-11.2
Eurytemora affinis	9.1; 7.5-13.7	8.1; 6.9-13.6	NS	NS
Limnocalanus macrurus	NS	NS	NS	150.2; 93.6-187.9
Pseudocalanus acuspes	NS	20.3; 18.4-35.9	NS	19.3; 14.6-28.5
	Cladocera			
Bosmina coregoni maritima	3; 1.5-3.8	2.3; 1.1-2.8	NS	NS
Cercopagis pengoi	31.7; 22.4-41.1	NS	NS	NS
