# Supplementary material for: Mercury-methylating bacteria are associated with copepods: A proof-of-principle survey in the Baltic Sea
Source: PLoS One. 2020 Mar 16;15(3):e0230310. doi: 10.1371/journal.pone.0230310 (PMC7075563; doi:10.1371/journal.pone.0230310)
Supplement: S2 Table — (PDF) [file pone.0230310.s003.pdf]

**S2 Table. Synthetic oligonucleotides used as standards for *hgcA* gene amplification.**

The single-stranded DNA includes a sequence between the forward and the reverse primers (underlined); *caatc* and *gaaca* (lowercase letters) were added at the 5' and 3'-end, respectively.

| Group | Species and strain | GenBank accession number | Sequence (5´→ 3´) | Length, bp |
|---|---|---|---|---|
| *Deltaproteobacteria* | *Desulfovibrio desulfuricans* ND132 | CP003220.1 | caatcGCCAACTACAAGCTGACCTTCGACACCCTGCGCGAGCGGCTGACCTCCATCGACGCCTGGCTGCTGGTGGATACGCGCGGCATCAACGTCTGGTGCGCGGCGGGgaaca | 117 |
| *Firmicutes* | *Desulfitobacterium metallireducens* DSM 15288 | CP007032.1 | caatcTGGACCAGTGAAAGCCAAAGATATCTCCGCGTTTATCAATTCAGGAATGAAAGCCACGGTAGAAATGCGGGCAGTCACATTTCATACATATGATCGATTAGTTTTAACTCCAGTTGAATTAGTCAATTCGCTCAAGGTTTCTCTAATTGATTTTTGGGATTCTCTTTTgaaca | 177 |
| *Archaea* | *Methanomethylovorans hollandica* DSM 15978 | CP003362.1 | caatcAACTACACGCTTAGCTTTGATGCTGTGCGCTTGCAGGTACTGACTGGTTATATCCTTGTGCTGGATACAAAGGGAATTAATGTATGGTGTGCTGCAGGCAAAGGCACATTCGGGACAGAgaaca | 129 |
