# Supplementary material for: Mercury-methylating bacteria are associated with copepods: A proof-of-principle survey in the Baltic Sea
Source: PLoS One. 2020 Mar 16;15(3):e0230310. doi: 10.1371/journal.pone.0230310 (PMC7075563; doi:10.1371/journal.pone.0230310)
Supplement: S3 Table — (PDF) [file pone.0230310.s004.pdf]

S3 Table. Summary of the primers in qPCR analysis for each group.

The corresponding size of the amplicon and primer concentration in the reaction are also shown.

**Group**	**Primer name**	**Sequence (5´→ 3´)**	**Amplicon length, bp**	**Concentration, nM**
Deltaproteobacteria	ORNL-Delta-HgcA-F	GCCAACTACAAGMTGASCTWC	107	250
	ORNL-Delta-HgcA-R	CCSGCNGCRCACCAGACRTT		250
Firmicutes	ORNL-SRB-Firm-HgcA-F	TGGDCCGGTDARAGCWAARGATA	167	250
	ORNL-SRB-Firm-HgcA-R	AAAAGAGHAYBCCAAAAATCA		250
Archaea	ORNL-Archaea-HgcA-F	AAYTAYWCNCTSAGYTTYGAYGC	119	500
	ORNL-Archaea-HgcA-R	TCDGTCCCRAABGTSCCYTT		250
