# Supplementary material for: Mercury-methylating bacteria are associated with copepods: A proof-of-principle survey in the Baltic Sea
Source: PLoS One. 2020 Mar 16;15(3):e0230310. doi: 10.1371/journal.pone.0230310 (PMC7075563; doi:10.1371/journal.pone.0230310)
Supplement: S4 Table — (PDF) [file pone.0230310.s005.pdf]

S4 Table. Standard curve parameters: cycle number, amplification efficiency and limit of quantification for each assay. Amplification efficiency (Eff.) and limit of quantification (LOQ; $n = 6$) were estimated with GenEx software (v. 6.1.0; *www.multiD.se*).

Clade	Template	DNA copy number						Eff.	LOQ
		1.5×10^2	1.5×10^3	1.5×10^4	1.5×10^5	1.5×10^6	1.5×10^7		
Deltaproteobacteria	*Desulfovibrio desulfuricans*	34.0 ± 0.4	30.3 ± 0.2	26.6 ± 0.1	23.0 ± 0.2	19.5 ± 0.1	16.2 ± 0.1	91	1.8×10^3
Firmicutes	*Desulfitobacterium metallireducens*	30.6 ± 0.2	27.3 ± 0.2	23.5 ± 0.1	20.1 ± 0.2	16.9 ± 0.1	13.7 ± 0.1	97	2.1×10^2
Archaea	*Methanomethylovorans hollandica*	>40.0	36.8 ± 0.3	32.3 ± 0.2	28.2 ± 0.1	25.2 ± 0.2	21.1 ± 0.1	82	6.9×10^4
