# Supplementary material for: Mercury-methylating bacteria are associated with copepods: A proof-of-principle survey in the Baltic Sea
Source: PLoS One. 2020 Mar 16;15(3):e0230310. doi: 10.1371/journal.pone.0230310 (PMC7075563; doi:10.1371/journal.pone.0230310)
Supplement: S5 Table — (PDF) [file pone.0230310.s006.pdf]

S5 Table. Amplification conditions for qPCR assays.

The conditions were adopted from the method of Christensen and co-workers; see reference list in the article.

Clade	Step 1	Step 2	Melt curve
Deltaproteobacteria	95 °C × 3 min	95 °C × 15 s, 65 °C × 20 s	65-95 °C, 0.5 °C/5 s
Firmicutes	95 °C × 3 min	95 °C × 10 s, 47 °C × 10 s, 58 °C × 60 s	58-95 °C, 0.5 °C/5 s
Archaea	95 °C × 3 min	95 °C × 30 s, 50 °C × 10 s, 55 °C × 60 s	55-95 °C, 0.5 °C/5 s
