# Supplementary material for: Mercury-methylating bacteria are associated with copepods: A proof-of-principle survey in the Baltic Sea
Source: PLoS One. 2020 Mar 16;15(3):e0230310. doi: 10.1371/journal.pone.0230310 (PMC7075563; doi:10.1371/journal.pone.0230310)

S1 Fig. Monitoring stations in the northern Baltic Proper, Åland Sea and Bothnian Sea used for zooplankton collections.

The stations F64 and US5b were visited by various cruises with R/V *Aranda* (SYKE, Finland) as a part of COMBINE cruises within he Finnish Baltic Sea monitoring programme. The station Landsort Deep (often referred to as BY31) by cruises with R/V *Fyrbryggaren* as a part of sampling within Swedish National Marine Monitoring Programme (SNMMP) and station H4 by trips with various smaller vessels within Himmerfjärden Eutrophication Study (http://www2.ecology.su.se/dbhfj/index.htm). The collection methods were identical and followed HELCOM guidelines (http://www.helcom.fi/action-areas/monitoring-and-assessment/manuals-and-guidelines/combine-manual).

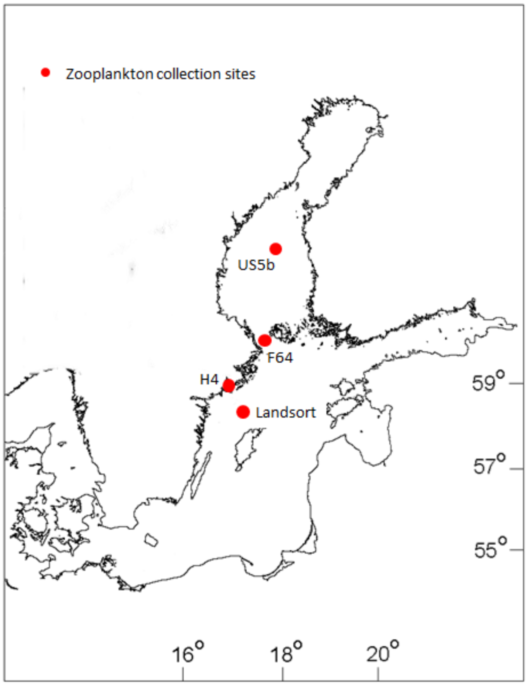

Supplement: S1 Fig — (PDF) [file pone.0230310.s008.pdf]
