# Supplementary material for: Mercury-methylating bacteria are associated with copepods: A proof-of-principle survey in the Baltic Sea
Source: PLoS One. 2020 Mar 16;15(3):e0230310. doi: 10.1371/journal.pone.0230310 (PMC7075563; doi:10.1371/journal.pone.0230310)

S2 Fig. Standard curves obtained with the templates for three clades carrying the *hgcA* gene.

The red line indicates the cutoff amplification cycle ($C_t = 30$) used for data analysis. $R^2 > 0.99$ and $n = 6$ in all cases; the error bars indicating SD are not visible. The solid black lines indicate average species- and site-specific range of the target concentrations in the test samples. In the regressions, *X* variable is Log_{10} (DNA copy number) and *Y* variable is Ct.

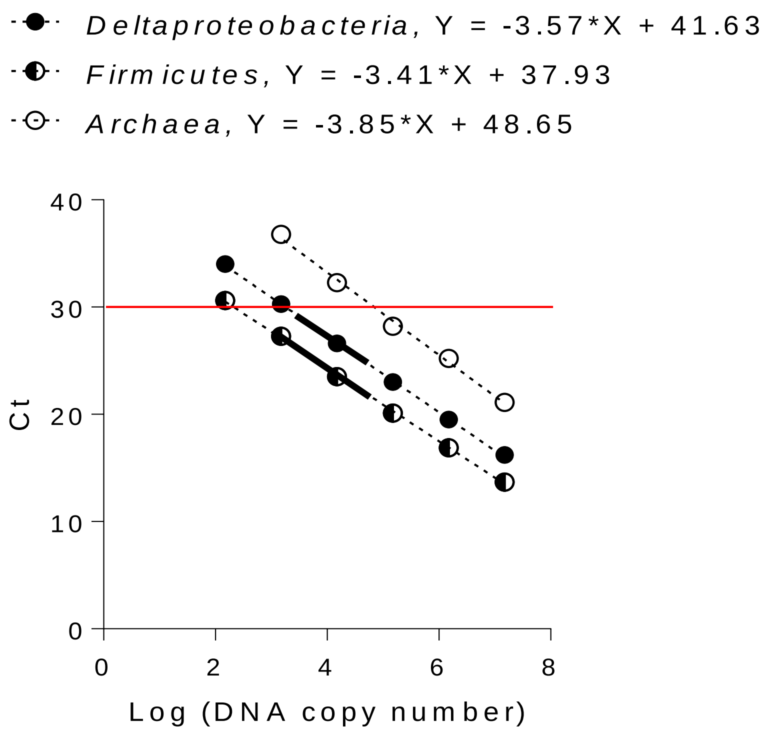

Supplement: S2 Fig — (PDF) [file pone.0230310.s009.pdf]
