# Supplementary material for: Mercury-methylating bacteria are associated with copepods: A proof-of-principle survey in the Baltic Sea
Source: PLoS One. 2020 Mar 16;15(3):e0230310. doi: 10.1371/journal.pone.0230310 (PMC7075563; doi:10.1371/journal.pone.0230310)

S3 Fig. Representative amplification plots and melt curves for three clades carrying the *hgcA* gene. Red line indicates the melt temperature. The cutoff amplification cycle (Ct = 30) was used for all assays. For clarity, the standards were removed from the plots.

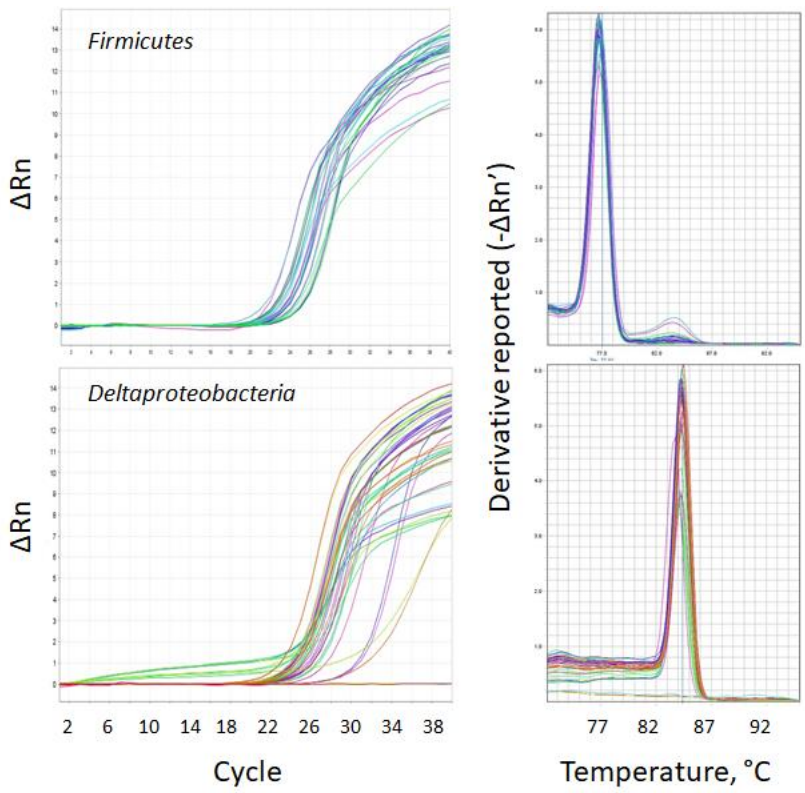

Supplement: S3 Fig — (PDF) [file pone.0230310.s010.pdf]
