# Supplementary material for: Mercury-methylating bacteria are associated with copepods: A proof-of-principle survey in the Baltic Sea
Source: PLoS One. 2020 Mar 16;15(3):e0230310. doi: 10.1371/journal.pone.0230310 (PMC7075563; doi:10.1371/journal.pone.0230310)
Supplement: Attachment — Submitted filename: Reply to Reviewers.pdf [file pone.0230310.s011.pdf]

Dear Editor, Dear Reviewers,

We appreciate all these excellent questions and comments, they were very helpful in revising the manuscript. Below are our answers and explanations, hopefully they are sufficient, but we can always elaborate if necessary. The replies are numbered consequently to facilitate cross-referencing, the line numbers refer to the revised version of the manuscript.

On behalf of the co-authors,

Elena Gorokhova.

**Reviewer #1**: This is a well written and concise manuscript describing the potential for mercury-methylation within zooplankton. This is potentially an important mechanism for understanding the overall mercury dynamics in aquatic food webs. The material is a bit limited making far-reaching conclusions difficult to draw. A more comprehensive study would have been achieved if authors would have waited with publication until more quantitative data was available. But they are clear about the limitations in the manuscript, and if the Editor agrees, this study could be published as a proof-of-concept study.

**Reply 1**: It is correct that a larger data set and a full screening for identification of the methylation markers are needed. However, doing the full analysis would imply a much larger project, both resource-wise and time-wise, and a different format of the paper. At this point, we believe that reporting occurrence of hgcA genes in the guts of zooplankton, which is an ecological group ubiquitously present in all aquatic food webs has a value of its own. It is our hope that this short report provides a strong indication that we should look for methylators in zooplankton guts as well as a useful description of the sample preparation for the analysis of resident bacteria in these animals (not a trivial task requiring gut cleaning from the bacteria present in the ingested detritus) that will stimulate other researchers worldwide to start collecting and examining local plankton communities.

Apart from this general comment I only have a few minor comments.
Line 89: Year seems to be missing in the reference.

**Reply 2**: Thank you, corrected.

Line 128: Please give rationale for selecting these clades.

**Reply 3**: Good point. The rationale has been presented in the Introduction and S1 Text, but, indeed, a short explanation makes it clearer. Added (**lines 140-142**).

Line 241: This section is speculative. Please make sure that you refer to this as a suggestion/hypothesis that needs further testing.

**Reply 4**: We modified the introductory sentence to point out that this suggestion is speculative (**line 263**).

**Reviewer #2**: Review of the manuscript PONE-D-19-31877 Mercury-methylating bacteria are associated with copepods: a proof-of-principle survey in the Baltic Sea, by Elena Gorokhova, Anne L. Soerensen and Nisha H. Motwani.

The manuscript deals with a very intriguing topic, that Hg-methylating microorganism associated with zooplankton gut microbiota might play a role in food-web transferring of MeHg. I found the topic very interesting from an ecological and ecotoxicology point of view. The manuscript is clear and well written and the experimental sections are robust. I only have some minor comments.

EXPERIMENTAL
Line 75: 'Zooplankton were collected…….'

Please provide the experimental details for plankton sampling and sorting.

**Reply 5**: Yes, thank you. The details on sampling are provided, **lines 83-87**. Please, observe that more details can be found in S1 Text file, Supporting Information.

Line 110: 'Reference samples used as a contamination control were hatched Artemia spp. ......'
Where Artemia used to test whether bacterial contamination occurred during the experimental procedure?

**Reply 6**: The contamination could have occurred during both the experimental procedured and sample handling. However, these reference samples also served as a control for false positive amplification originating from both *Rhodomonas* and (presumably) hgcA-free animal gut. This has been clarified; **lines 125-128**.

qPCR assay
Line 134: ……..1.5×106 to 1.5×102 apparent copies of target DNA per reaction.
Why the DNA concentration is expressed as apparent copies of target DNA and not as ng/µl? Did the authors loaded a known copy number of synthetic DNA oligonucleotides per reaction?

**Reply 7**: Yes, using nominal number of copies per reaction is exactly the point with a synthetic standard. This is explained in detail in the reference provided (Vermeulen et al., 2009). See also S1 Text file.

Discussion
As the authors discussed the importance of fecal pellets for Hg methylation, I was wondering whether there was any visible fecal material in the copepod gut during feeding on Rhodomonas salina and if these could have affected the results.

**Reply 8**: The reddish coloration of the guts was used as an indication of the gut depuration from the prey collected in the field. Thus, the Rhodomonas was used to purge the food bolus from zooplankter guts and remove any potential hgcA-containing microorganisms associated with detritus and other food items retaining only microbes closely associated with the gut mucosa.

This was the desired effect. See also Reply 6, if the question refers to a possible contamination of the gut bolus via Rhodomonas culture.
