# Supplementary material for: Mercury-methylating bacteria are associated with copepods: A proof-of-principle survey in the Baltic Sea
Source: PLoS One. 2020 Mar 16;15(3):e0230310. doi: 10.1371/journal.pone.0230310 (PMC7075563; doi:10.1371/journal.pone.0230310)
Supplement: Attachment — Submitted filename: reply to Reviewer.pdf [file pone.0230310.s012.pdf]

Dear Editor, Dear Reviewer,

We appreciate all comments and suggestions. They were very helpful in revising the manuscript. Below are our answers and explanations, hopefully they are sufficient, but we can always elaborate, if necessary. The replies are numbered consequently to facilitate cross-referencing, the line numbers in the comments refer to the line numbering in the original version and those in the replies – in the revised version of the manuscript.

On behalf of the co-authors,

Elena Gorokhova.

Reviewer #3: General comments

The authors present an interesting story about potential for mercury-methylating bacteria in copepods, including a small sample set analyzed with previously published primers. The authors go on to speculate what the data might mean in the context of mercury distributions in the Baltic Sea.

> **Reply 1:** Thank you. We hope that that our study would stimulate a more system-specific research addressing endogenous methylation capacity of aquatic microbiomes. Although our findings are largely descriptive, more focus on experimental studies measuring methylation capacity in concert with molecular identification of the genes and taxonomic groups involved will provide knowledge on the methylation pathways that are not currently considered in mercury biogeochemistry.

The story is interesting, but my main concern is the specificity of the qPCR assays. What evidence do the authors have that these primers don't amplify genomic DNA from copepods, or from some other microbial targets? Was the zooplankton food (Rhodomonas) tested for these targets?

> **Reply 2:** We fully agree that assay specificity is important. And, of course, it would be impossible to guarantee that no mispriming occurred in any of the field samples. However, the primers for clade-specific identification of hgcAB genes were extensively tested against a broad set of cultures with published genomes during the last years (Podar et al. 2015; Christensen et al. 2016; McDaniel et al. 2020). Although the diversity of microorganisms capable of methylation has been greatly expanded in recent years (Jones et al. 2019; McDaniel et al. 2020), no mispriming for these group-specific primers has been reported, and no positive amplification has been reported for eukaryotic DNA. *In silico* studies also exclude this possibility (Podar et al. 2015). Therefore, the hgcAB cluster and group-specific primers (not the universal primers!) are considered a reliable molecular marker to identify mercury methylating microbial communities and predict MeHg production in the environment (Bravo and Cosio; Tang et al. 2020).
>
> As far as we know, no mixed community studies aiming at identification of Hg-methylating capacity and the microorganisms involved have experimentally addressed a possibility that eukaryotic DNA might contribute to hgcAB gene copy number DNA extracted from soil or sediment (Compeau and Bartha 1985; Schaefer et al. 2014; Christensen et al. 2017; Ma et al. 2019), periphyton communities (Schaefer et al. 2014; Olsen et al. 2016; Dranguet et al. 2018), and microbiome of some terrestrial arthropods (Podar et al. 2015) because of the high specificity of these group-specific primers.
>
> Having said this, we believe that we have explored all key sources of non-specific amplification that were feasible and meaningful. The following measures were taken:
>
> - First, zooplankters retrieved from the net were placed in 0.2-µm filtered aerated seawater and supplied with an excess of axenic *Rhodomonas* to clear the guts of any potential hgcA-containing microorganisms associated with their food items and only

retain those microbes closely associated with the gut mucosa. It should be noted, however, that transitory microflora (if, in spite of the gut cleaning procedure, these microorganisms had contributed to the hgcA yield) may be as important for the overall methylating capacity of the gut as the stationary microflora.

- Second, the dissected guts (and not the whole animals) were used for DNA extraction, thus minimizing the contribution of host DNA in the qPCR assay. This is important, because the inhibitory effect of the host DNA (if any) should be proportional to its amount in the reaction.
- Third, samples of *Artemia* guts filled with *Rhodomonas* (i.e., reference samples) were used as a procedural control for (i) contamination, and (ii) non-specific amplification by the algal DNA. When setting the protocol for these experiments, filter-collected *Rhodomonas* was also tested and found negative. It was not, however, meaningful to include the DNA extracted from the filters in the method description here, because our reference samples served both purposes (i.e., contamination and negative amplification from the food itself).
- Fourth, prompted by this comment, in addition to the spike test with cladoceran DNA, we conducted an additional test using copepod samples that were tested negative for Archaeal hgcA genes. The outcome is now included to Supporting Information (S1 Text [Spiking test]). Note that using whole bodies is not particularly meaningful, because (1) hgcA-carrying bacteria can be present in the microbiome communities of the carapace, and (2) host DNA will heavily dominate the template. This is why the spiking tests for copepods were restricted to the negative samples for the assays with Archaea primers. For a proper evaluation, one would need copepod samples that are free from any hgcA-bearing microorganisms.
- Fifth, the melt-curve analysis was conducted for all samples and confirmed occurrence of a single peak in all cases. In the revised version, representative melt-curve plots were added as S3 Fig (Supporting Information).
- Finally, we applied a routine spiking test (one for each copepod species) with a standard at the lowest dilution (one for Deltaproteobacteria and one for Firmicutes) and confirming that amplicon produced a single peak in the melt curve.

Given these methodological precautions and tests, it would be extremely unlikely that the data generated by the qPCR assays were erroneous due to a non-specific amplification.

Were the actual data provided with the manuscript (raw data showing data for all replicates etc.) I did not see them.

Reply 3: Very good point, thank you. The primary data and the data behind Figure 1 are now provided as S6 Table, Supporting Information.

The text has a few issues with grammar and style. I have indicated some examples of these issues in my review below.

Reply 4: Much appreciated, we have considered all comments and made changes when necessary.

Specific comments

L11 food-webs >food webs

Reply 5: Yes, corrected.

L12-14 Sentence should be reworded. It seems you refer to the sediments and detritus as a ‘source’, but it is unclear what product originates from ’the source’; the wording should be revised for clarity.

Reply 6: We were not sure if we understood this comment, but reworded the sentence to avoid ambiguity.

L15 Baltic > Baltic Sea

Reply 7: “Baltic zooplankton” as well as “Baltic fish” are commonly used expressions and we confirmed with a proof—reading service that it is grammatically acceptable. However, we reworded this sentence to point out that this zooplankton was dominant in the Northern Baltic Sea (not generally in the entire sea).

L38 in a some > in some

Reply 8: Corrected, thank you.

L43 I suggest deleting ‘thus’ here, as the sentence seems independent from the prior sentence.

Reply 9: Corrected, thank you.

L64 Baltic>Baltic Sea

Reply 10: See Reply 7. Rephrased anyway.

L95 Was the Rhodomonas culture axenic? Did you test the presence of MeHg genes in the culture?

Reply 11: Yes, the culture was axenic (stated in M&M, line 122) and it was confirmed that it is hgcA-free. See Reply 2.

L139 The authors heavily cite previous studies on the qPCR method. Some more detail on qPCR approach should be provided. State that the assay was a SYBR assay and explain how you determined whether there was non-target amplification (i.e. explain results from melt curves).

Reply 12: As the qPCR protocol was adopted from the study of Christensen and co-workers (Christensen et al. 2016), it was presented only briefly. However, we presented details for our synthetic standards as these are not available from the originally described protocol, where bacterial cultures were used to construct standard curves. All other information relevant for the qPCR assay is provided as Supporting Information (Laboratory procedures, S2-S5 Tables and S2 Figure. We agree, however, that is was necessary to mention that the assay was a SYBR assay, and this information is now added to M&M (line 144).

A figure showing examples of melt curves was added to Supporting Information (S3 Fig). In the M&M section, a line was added to clarify that for all amplicons, only one peak was detected (line 153). See also Reply 2.

The DNA extraction method seems fairly ‘dirty’ as it does not include Proteinase nor a purification step beyond the Chelex step.

Reply 13: The purity of DNA in our samples was acceptable as indicated by the mean A260/280 ratio (1.78; range: 1.64-1.85) as specified in S1 Table. In our hands, Chelex protocol provides the best yield for extracting bacterial DNA from crustacean (i.e., chitin-rich) samples. Moreover, we tested the usefulness of the proteinase step and found that it decreases yield of bacterial DNA. We have successfully applied the Chelex method for both qPCR assays and sequencing in the analysis in molecular diet and microbiome (Gorokhova 2009; Gorokhova and Engström-Öst 2009; Edlund et al. 2012; Motwani and Gorokhova 2013; Gorokhova et al. 2015; Motwani et al. 2018; Motiei et al. 2020). We have also recently used Chelex for extracting genomic DNA from a crustacean for mass-spectrometry analysis (Gorokhova et al. 2020) and found that it outperformed several commercial kits.

Did you conduct inhibition tests with the copepod DNA (see my comment on the supplementary text)?

Reply 14: Prompted by this comment, we conducted additional tests and provided the results in Supporting Information (S1 Text [Spiking test]). In short, no significant inhibition was observed for either cladoceran or copepod samples. For cladoceran samples, the inhibition tests were motivated by the fact that no amplification was observed. As all samples with copepod guts produced positive amplification for Deltaproteobacteria and Firmicutes, it was not very relevant to test the inhibition by copepod DNA, especially considering that the exact copy number of hgcA genes was difficult to infer (see lines 160-166). However, the lack of amplification for Archaea in these samples might, in theory, be related to a complete inhibition. Therefore, we acknowledged that this test was necessary and added it to the spiking experiments.

L156-157 Here you assume that there is one gene copy per cell and one genome per cell. Some justification for this is needed. I would avoid speculating this far with qPCR data, especially with all the uncertainties highlighted by the authors.

Reply 15: Please, observe that this assumption was based entirely on the work by Christensen and co-workers, who measured it in a limited number of bacterial strains (Christensen et al. 2016). Of course, this cannot be extrapolated for the mixed communities, but this is the best available knowledge at the moment. We clarified the origin of this assumption in the revised manuscript (lines 161-162).

L159 Detection limit of 10^6 is alarmingly high for one of the assays (essentially means a non-detect as such amplification could easily occur with non-targets). The test efficiency of 60% is also alarmingly low, suggesting poor match with target and primers, or degraded standards or primers.

Reply 16: Please, observe that detection limit of 10^6 and amplification efficiency of 60% were reported for some strains by Christensen and co-workers; these are not what we observed in our assays (S4 Table). We revised the text to avoid misunderstanding (lines 161-164).

The authors may consider changing the names for the primers or specifying what they actually amplify.

Reply 17: The primer names were adopted from the original method paper (Christensen et al. 2016) and changing these names would be confusing; S2 and S3 Tables describe the sequences that were amplified.

L166 were tested > tested

Reply 18: Corrected, thank you.

L171-173 Sentence should be reworded for clarity. The verb is very far from subject in the sentence which makes it very difficult to follow. I also don't follow what you mean by 'indicative'. Indicative of presence of mercury methylators? Please reword for clarity.

Reply 19: The sentence was confusing, indeed. Revised (lines 175-177), thank you.

L176 lack of amplification of archaeal Hg-methylators?

Reply 20: Yes, the sentence is completely reworded to clarify that (lines 179-180).

L176-177 The sentence should be reworded for accuracy. There wasn't a difference in amplification efficiency among bacteria - the difference was among the qPCR assays targeting different bacterial Hg-methylation genes.

Reply 21: The sentence was revised to avoid confusion (lines 181-182). The difference is question was difference among bacterial strains using in the same qPCR assays as reported by Christensen and co-workers.

Fig 1. Caption. Baltic>Baltic Sea

Reply 22: Corrected.

L185 You state that n=3-4; however, each sample was pooling many guts, correct? It would be helpful to remind the reader about it here.

Reply 23: Yes, this information is added to the figure caption (lines 191-192).

L187 and 191 delete: 'positive' here and elsewhere where it appears with 'amplification'

Reply 24: Thank you, done.

L192-193 The figure x-axis explanation is confusing. It sounds like you are saying that absence of a species on the x-axis means there were no individuals of that species collected at that station; thus, it would follow that there were no zeros (no undetects) among the samples. Are you really meaning to say that in each case you ran qPCR with copepod DNA you always detected the targets? Please clarify.

Reply 25: There was no undetects, correct. Considering that in each sample, 25 to 30 guts were pooled, this is not particularly surprising.

L220-221 The sentence is confusing. I suggest breaking it into two to separately state what you mean by copy number differences and how this links with phylogenetic differences. Do you mean phylogenetic differences of the microbiomes or the zooplankton?

Reply 26: The sentence was revised as suggested (lines 224-227). Phylogenetic differences were referring to zooplankton (copepods vs. Cercopagis).

L222 yielded

Reply 27: Corrected.

S2 Figure

Include the amplification curve equations in the figure.

Reply 28: Included.

S4 Table

Write out in full the names of target organisms. Include the strain identifiers.

Reply 30: Done.

S1 text. QPCR 'setup'

The citations are inconsistent. At least in one case, a citation is included as a superscript, not in parentheses.

Reply 31: Corrected and checked for inconsistencies.

You state that you added 6 uL of DNA onto the wells, then 'dried' them for 30 min. This is an unusual step and should be explained.

Reply 32: This step has been adopted together with the entire protocol. The purpose of the evaporation step is to decrease the sample volume.

You discuss melt curves here – explain what they showed and how it influenced what data were included. If there were several peaks the data can't be used.

Reply 33: The melt curves have been added as S3 Figure. Only single peaks within each assay were observed. See Reply 2.

Spike test

Here you explain a spike test conducted with the cladoceran DNA. Did you conduct a similar test with the copepod DNA to confirm there was no inhibition?

Reply 34: The spike test for the copepod samples has been added. See Reply 2.

References

Bravo, A. G., and C. Cosio. Biotic formation of methylmercury: A bio–physico–chemical conundrum. Limnol. Oceanogr. **in press.** doi:10.1002/lno.11366

Christensen, G. A., A. C. Somenahally, J. G. Moberly, and others. 2017. Carbon Amendments Alter Microbial Community Structure and Net Mercury Methylation Potential in Sediments. Appl. Environ. Microbiol. **84**: e01049-17. doi:10.1128/AEM.01049-17

Christensen, G. A., A. M. Wymore, A. J. King, and others. 2016. Development and Validation of Broad-Range Qualitative and Clade-Specific Quantitative Molecular Probes for Assessing Mercury Methylation in the Environment. Appl. Environ. Microbiol. **82**: 6068–6078. doi:10.1128/AEM.01271-16

Compeau, G. C., and R. Bartha. 1985. Sulfate-Reducing Bacteria: Principal Methylators of Mercury in Anoxic Estuarine Sediment. Appl. Environ. Microbiol. **50**: 498–502.

Dranguet, P., V. I. Slaveykova, and S. L. Faucheur. 2018. Kinetics of mercury accumulation by freshwater biofilms. Environ. Chem. **14**: 458–467. doi:10.1071/EN17073

Edlund, A., K. Ek, M. Breitholtz, and E. Gorokhova. 2012. Antibiotic-Induced Change of Bacterial Communities Associated with the Copepod Nitocra spinipes. PLoS ONE **7**. doi:10.1371/journal.pone.0033107

Gorokhova, E. 2009. Toxic cyanobacteria Nodularia spumigena in the diet of Baltic mysids: Evidence from molecular diet analysis. Harmful Algae **8**: 264–272. doi:10.1016/j.hal.2008.06.006

Gorokhova, E., and J. Engström-Öst. 2009. Toxin concentration in *Nodularia spumigena* is modulated by mesozooplankton grazers. J. Plankton Res. **31**: 1235–1247. doi:10.1093/plankt/fbp060

Gorokhova, E., G. Martella, N. H. Motwani, N. Y. Tretyakova, B. Sundelin, and H. V. Motwani. 2020. DNA epigenetic marks are linked to embryo aberrations in amphipods. Sci. Rep. **10**. doi:10.1038/s41598-020-57465-1

Gorokhova, E., C. Rivetti, S. Furuhagen, A. Edlund, K. Ek, and M. Breitholtz. 2015. Bacteria-Mediated Effects of Antibiotics on Daphnia Nutrition. Environ. Sci. Technol. **49**: 5779–5787. doi:10.1021/acs.est.5b00833

Jones, D. S., G. M. Walker, N. W. Johnson, C. P. J. Mitchell, J. K. C. Wasik, and J. V. Bailey. 2019. Molecular evidence for novel mercury methylating microorganisms in sulfate-impacted lakes. ISME J. **13**: 1659–1675. doi:10.1038/s41396-019-0376-1

Ma, M., H. Du, T. Sun, S. An, G. Yang, and D. Wang. 2019. Characteristics of archaea and bacteria in rice rhizosphere along a mercury gradient. Sci. Total Environ. **650**: 1640–1651. doi:10.1016/j.scitotenv.2018.07.175

McDaniel, E. A., B. Peterson, S. L. R. Stevens, P. Q. Tran, K. Anantharaman, and K. D. McMahon. 2020. Expanded Phylogenetic Diversity and Metabolic Flexibility of Microbial Mercury Methylation. bioRxiv. doi:10.1101/2020.01.16.909358

Motiei, A., B. Brindefalk, M. Ogonowski, and others. 2020. Disparate effects of antibiotic-induced microbiome change and enhanced fitness in Daphnia magna. PLOS ONE **15**: e0214833. doi:10.1371/journal.pone.0214833

Motwani, N. H., J. Duberg, J. B. Svedén, and E. Gorokhova. 2018. Grazing on cyanobacteria and transfer of diazotrophic nitrogen to zooplankton in the Baltic Sea. Limnol. Oceanogr. **63**: 672–686. doi:10.1002/lno.10659

Motwani, N. H., and E. Gorokhova. 2013. Mesozooplankton Grazing on Picocyanobacteria in the Baltic Sea as Inferred from Molecular Diet Analysis E. Sotka [ed.]. PLoS ONE **8**: e79230. doi:10.1371/journal.pone.0079230

Olsen, T. A., C. C. Brandt, and S. C. Brooks. 2016. Periphyton Biofilms Influence Net Methylmercury Production in an Industrially Contaminated System. Environ. Sci. Technol. **50**: 10843–10850. doi:10.1021/acs.est.6b01538

Podar, M., C. C. Gilmour, C. C. Brandt, and others. 2015. Global prevalence and distribution of genes and microorganisms involved in mercury methylation. Sci. Adv. **1**: e1500675. doi:10.1126/sciadv.1500675

Schaefer, J. K., R.-M. Kronberg, F. M. M. Morel, and U. Skyllberg. 2014. Detection of a key Hg methylation gene, *hgcA* , in wetland soils: Detection of the Hg methylation gene, hgcA, in soils. Environ. Microbiol. Rep. **6**: 441–447. doi:10.1111/1758-2229.12136

Tang, W.-L., Y.-R. Liu, W.-Y. Guan, H. Zhong, X.-M. Qu, and T. Zhang. 2020. Understanding mercury methylation in the changing environment: Recent advances in assessing microbial methylators and mercury bioavailability. Sci. Total Environ. **714**: 136827. doi:10.1016/j.scitotenv.2020.136827
